# Dynamics of the Prefrontal Cortex during Chess-Based Problem-Solving Tasks in Competition-Experienced Chess Players: An fNIR Study

**DOI:** 10.3390/s20143917

**Published:** 2020-07-14

**Authors:** Telmo Pereira, Maria António Castro, Santos Villafaina, António Carvalho Santos, Juan Pedro Fuentes-García

**Affiliations:** 1 Polytechnic Institute of Coimbra, Coimbra Health School, 3046-854 Coimbra, Portugal; telmo@estescoimbra.pt (T.P.); mac@estescoimbra.pt (M.A.C.); acs@estescoimbra.pt (A.C.S.); 2 Centre for Mechanical and Engineering Materials and Processes, University of Coimbra, 3030-788 Coimbra, Portugal; 3 Faculty of Sport Science, University of Extremadura. Avda: Universidad S/N, 10003 Cáceres, Spain; jpfuent@unex.es

**Keywords:** chess, prefrontal cortex, functional near-infrared spectroscopy

## Abstract

This study aimed to compare the dynamics of the prefrontal cortex (PFC), between adult and adolescent chess players, during chess-based problem-solving tasks of increasing level of difficulty, relying on the identification of changes in oxygenated hemoglobin (HbO2) and hemoglobin (HHb) through the functional near-infrared spectroscopy (fNIRS) method. Thirty male federated chess players (mean age: 24.15 ± 12.84 years), divided into adults and adolescents, participated in this cross-sectional study. Participants were asked to solve three chess problems with different difficulties (low, medium, and high) while changes in HbO2 and HHb were measured over the PFC in real-time with an fNIRS system. Results indicated that the left prefrontal cortex (L-PFC) increased its activation with the difficulty of the task in both adolescents and adults. Interestingly, differences in the PFC dynamics but not in the overall performance were found between adults and adolescents. Our findings contributed to a better understanding of the PFC resources mobilized during complex tasks in both adults and adolescents.

## 1. Introduction

The prefrontal cortex (PFC) has been thoroughly described as the center of cognitive function, being involved in executive functions that include decision-making and problem-solving, also taking part in attention, memory, planning, motor control, and cognitive flexibility [1,2,3]. The PFC, also called the frontal associative cortex and the Magister of the mind [4], is heavily interconnected with other brain regions, receiving quite diverse sensory and cognitive inputs based on which overall coordination of behavior is implemented. Thus, this brain region, particularly the dorsolateral part, is responsible for the temporal organization of behavior, language, and reasoning [5], and the definition and coordination of plans for action [6] entailing its conceptualization and flexibility to the environmental demands [7]. Furthermore, emotional and inhibitory control processes have been associated with the orbitofrontal region of the PFC, while the medial region has implications in motivation and behavioral drive [5].

Several studies have demonstrated the importance of the frontal lobe, and particularly the PFC, in problem-solving tasks [8,9] including playing chess games [10]. Playing Chess is a particular and challenging activity that requires the orchestration of diverse cognitive resources such as memory, attention, and perceptual grouping [11]. It also involves the recognition of complex spatial relationships as determined by the game rules, and thus, the need to simultaneously handle multiple objects under such rule constraints [12]. In addition, motor timing, movement selection, and gait control are also enrolled in the multi-componential processes involved in chess-playing, all facets under PFC control [13]. Other studies have looked into the overall pattern of brain activation as a function of the level of expertise, identifying differences in the cortical resources engaged during chess-playing activities, with the experts manifesting significant activation of areas related to object perception or expertise-related pattern recognition [14,15], as well as recruitment in brain areas involved in knowledge storage and retrieval and memory, whilst the novice players activate predominantly brain areas involved in learning and retrieving of new information [10]. Higher activation of brain regions involved in attention and problem-solving was also demonstrated in expert chess players engaging a chess-based problem-solving task [16], highlighting the existence of significant differences in brain dynamics, and underlying cognitive operations in chess players.

The PFC is also of great interest in adolescence due to its relation to cognitive control and emotion processing [17]. Differences between adult and adolescence PFC have been reported, identifying a reduction in the gray matter between adolescence and adulthood [18]. This indicates that during adolescence, the prefrontal regions are still developing [19]. In this regard, the lateral regions of the PFC are the latest developing areas involved in executive regions [20]. A recent study examined the brain electrical pattern of adolescent chess players during problem-solving tasks [21]. However, this study did not compare the brain processing of adolescent and adult chess players.

Much of the available evidence concerning brain activation during chess playing tasks have been based on fMRI and electrophysiological methods, but to the best of our knowledge, no studies have previously addressed the PFC activation associated with chess playing tasks with a functional near-infrared spectroscopy (fNIRS) method. This method provides information on hemodynamic changes associated with cortical activation by noninvasively measuring changes in the relative ratios of deoxygenated hemoglobin (HHb) and oxygenated hemoglobin (HbO2) [22]. Comparatively to other non-invasive neuroimaging methodologies, fNIR is more tolerant to motion artifacts and provides a balance between spatial and temporal resolution, thus being a good method for tasks involving motor components [23,24].

Previous studies have compared the dynamics of the PFC between adults and adolescents using emotional tasks [25,26,27]. However, to the best of our knowledge, these comparisons have not been performed using high cognitive demand tasks such as chess. This would be relevant as it would allow reporting whether the differences in the PFC between adolescents and adults [18] have any impact on the dynamics of the PFC or in the task performance during high demand cognitive tasks. Hence, we sought to compare the dynamics of the PFC activation during three chess-based problem-solving tasks of increasing level of difficulty in both competitive adult and adolescent chess players, relying on the identification of changes in HbO2 and HHb. To the best of our knowledge, this is the first experimental approach of the PFC activation in such particular challenging chess tasks monitored with fNIR, and, therefore, the results could contribute to a better understanding of the PFC resources mobilized during the handling of complex problems associated with chess playing in both adults and adolescents.

## 2. Materials and Methods

### 2.1. Study Design and Participants

Federated players from official Portuguese chess clubs were invited to participate in a cross-sectional study. The playing level for chess was determined by the ELO rating system, developed by Arpad Elo and introduced by the World Chess Federation (FIDE) as a ranking system [28]. It is a method for calculating the relative skill levels of players in competitor-versus-competitor games [29]. All the participants were classified according to the ranking system of the FIDE. Exclusion criteria included (1) inability to perform the tasks with the computer, (2) diseases that affect the autonomic and central nervous system, (3) not being on medication, and (4) not being classified by the International Chess Federation with ELO. A total of 31 players was selected to participate (30 males; 1 female) and screened for suitability based on clinical history, behavioral profile, and chess practice characterization. The female player was excluded to avoid gender bias. All the remaining volunteers met the research requirements and were included in the study, thus, a total of 30 male chess players (24.15 ± 12.84 years) were enrolled, with more than 4 years of continuous competitive chess playing experience (participation in chess competition on average: 10.79 ± 7.73 years). Half of the study population were adults (age > 19 years) and half were adolescents. The participants all had normal or corrected-to-normal vision. After a detailed description of the objective and research methodology, all participants signed an informed consent. The study was conducted according to the guidelines of the Declaration of Helsinki. Anonymity and confidentiality of the collected data were assured, and the study was developed for scientific purposes only, free of any financial or economic interests. All procedures were approved by the University Research Ethics committee (approval number: 85/2015).

### 2.2. Procedure

Data collection was made in an appropriate room, with adequate temperature and humidity, in a dimmed environment where light would not contaminate the collected information, and silent, so the participant’s concentration was not disturbed during the tests. Before the tests, a structured questionnaire was filled with sociodemographic information and details regarding the chess playing history, including years of practice, years of competitive playing, hours of practice per day and days of practice per week, and habit of playing in digital chess platforms and solving problems. The individual ELO score was calculated [28,29]. The participants were questioned about their baseline level of motivation towards the task and the degree of tiredness, providing such information on a 10-point scale.

The participants were then instructed on how to perform the tasks and all the requirements to ensure the quality of the physiological information and the ecological approach of the chess-based problem-solving tasks. The participants were seated in a comfortable chair in front of a computer screen that ran the chess problems and were monitored with a 16-channel fNIRS stand-alone functional brain imaging system (fNIR100A-2, Biopac System Inc., CA, USA), adjusted on the forehead and insulated using a dark light-proof tape. The fNIRS acquisition was performed with a dedicated computer running the COBI Studio program [30]. Real-time monitoring of HbO2 and HHb in the prefrontal cortex were performed while the participant solved each one of the three chess problems randomly presented. After each problem-solving task, the participants were inquired how they perceived the task in terms of complexity, difficulty, and level of engagement stress.

### 2.3. Chess Problems

**Figure 1:**
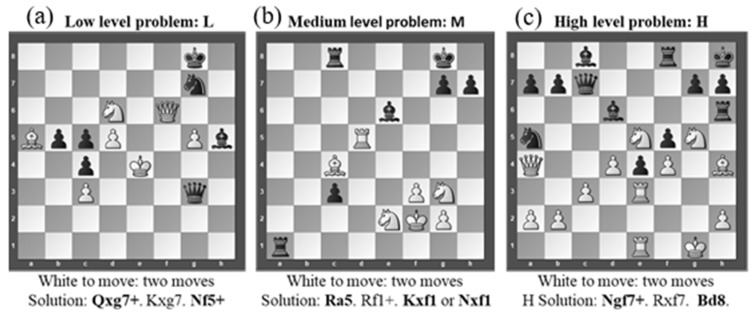
Representation of the three chess-based problem-solving tasks by level of complexity: (**a**) panel—low level problem (L); (**b**) panel—medium level problem (M); (**c**) panel—high level problem (H).

Before starting the experimental task, procedures and protocol requirements were explained to the participants. Moreover, all participants underwent a familiarization period with the computer and the equipment required for testing. Participants conducted a total of three chess-based problem-solving tasks. The problem-solving tasks were selected from Total Chess Training CT-ART 3.0 (Convekta, Moscow, Russia) by a FIDE master (ELO rating of 2300 or more). These chess problems consisted of three levels of difficulty intended for chess players with an ELO rating of 1600–2400 raised by Blokh [31], with 1 being the lowest and 10 the highest level of difficulty: low-level (1), medium-level (5), and high-level (10) chess problems. Participants had two and a half minutes to solve each problem. Two moves for each problem were required (see Figure 1).

The Fritz 15 software, using Stockfish 6, 64 BIT, for Windows was used as chess engine [32]. It is one of the strongest chess engines in the world and it is open source (GPL license). In addition, chess engines are a useful tool for chess training, being similar to the tactical responses given by humans [33]. A laptop was employed (Intel Core i7-6500U, (Intel, Santa Clara, USA) 1 TB, 8 GB memory DDR3L-SDRAM, (Dell, Round Rock, USA)). In order to simulate a real chess environment, the Fritz software automatically responded to moves with the best move previously computed by the research group, simulating a real chess environment. The research technician selected the Fritz level according to the ELO level of each player. This methodology was used in previous studies [34,35,36].

### 2.4. Functional Brain Imaging—fNIRS

**Figure 2:**
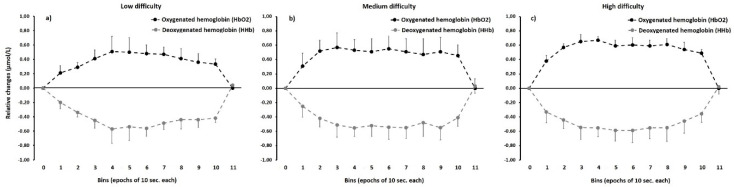
Example of the relative changes in oxygenated hemoglobin and deoxygenated hemoglobin in one participant during the three experimental tasks. Bin 0 marks the baseline and Bin 11 the end of each task. The relative changes were computed as the mean change in the overall optodes. (**a**) panel—low level problem (L); (**b**) panel—medium level problem (M); (**c**) panel—high level problem (H).

The measurement of prefrontal cortex activity was performed with an fNIRS system (fNIR100A-2, Biopac System Inc., CA, USA), as previously stated, which detects changes in HbO2 and HHb (both in μmol/L) resulting from brain activation [22,23,24]. Signal acquisition was performed using a sensor pad containing 4 light sources (LED) and 10 light detectors with a fixed source-detector distance of 2.5 cm and a depth of light penetration of approximately 1.5 cm beneath the scalp, generating an array of 16 measurement sites (voxels or channels) per wavelength [22,23,24]. The sensor array is embedded in a flexible pad and is placed over the forehead during signal acquisition. The light sources emit two infrared light wavelengths (730 nm and 850 nm) for every 16 channels. As the light penetrates the scalp, part of it is absorbed by the hemoglobin and the remaining light reaches the detectors in a banana-shaped path. Thus, concentrations of hemoglobin are calculated by the ratio of light absorbed at different wavelengths, considering that HbO2 and HHb have different absorption coefficients. The sampling rate of the system was 2 Hz and LED current and detector gain was adjusted prior to the acquisition to prevent signal saturation. The fNIRS acquisition was made for each problem-solving task, resulting in one signal file for each problem. The acquisition started with an initial 10-s baseline recording, after which the task was initiated, and was managed with a dedicated laptop running the Cognitive Optical Brain Imaging (COBI) Studio program [23] (Biopac system Inc., CA, USA). Figure 2 represents an example of the mean changes in HbO2 and HHb recorded for one participant during the time course of the three experimental tasks.

### 2.5. Data Processing

After visual inspection and elimination of low-quality channels and motion artifacts, the raw files were filtered with a 20-order low-pass finite impulse response (FIR) filter (0.02–0.40 Hz) and a cutoff frequency set at 0.1 Hz to remove long-term drift [30], high-frequency noise, and cardiac and respiratory cycle effects [23,24]. After this process, a sliding-window motion artifact rejection algorithm was used to filter out spikes and to improve signal quality [23]. Relative changes in the concentration of HbO2 (ΔHbO2) and HHb (ΔHHb) were calculated based on the modified Beer–Lambert law [23,24,37], with a 10-s baseline recorded at the beginning of each task. Blood oxygenation (Δoxy) was calculated as the difference of ΔHbO2–ΔHHb. Blood volume changes (ΔHbT) were calculated as ΔHbO2 + ΔHHb.

All aspects of data processing were managed with the fNIR software [23,24,30], version 4.5 (Biopac system Inc., USA). The absolute values obtained after data processing were Z-scored and outliers were removed [37]. Data per channel was averaged for each condition and four regions of interest (ROI) for the prefrontal cortex were created, as previously proposed [13], by grouping anatomically congruent channels. The generated ROIs were the left prefrontal cortex (L-PFC), the right prefrontal cortex (R-PFC), the left medial prefrontal cortex (LM-PFC), and the right medial prefrontal cortex (RM-PFC). For studying laterality effects, we further considered the mean left hemisphere prefrontal cortex (LH-PFC) and the mean right hemisphere prefrontal cortex (RH-PFC) by grouping channels accordingly.

### 2.6. Statistical Analysis

Data was gathered in Excel 2016 (Microsoft Office, Redmond, WA, USA), and then imported to SPSS Statistics version 24 (IBM, Armonk, NY, USA) for statistical analysis.

Categorical variables were reported as frequencies and percentages, and *χ*2 or Fisher exact tests were used when appropriate. The Shapiro–Wilks test was used to confirm the normal distribution of all continuous variables, expressed as mean and standard deviation. As stated before, the fNIRS variables were Z-scored. Other continuous variables with a non-normal distribution were log-transformed. Student’s *t*-test was applied for group comparisons of descriptive variables only. Individual variables were checked for homogeneity of variance via Levene’s test. A repeated-measures ANOVA was used to evaluate modifications of the variables between the three problem-solving tasks, in the whole population, in each, and between groups. Factors included in the ANOVA were task (three increasing levels of difficulty) and ROI (four prefrontal cortex locations, as described previously). Group was also entered (two levels: adults and adolescents) to test for interactions. The Greenhouse–Geisser correction was used when sphericity was violated, and the Bonferroni adjustment was adopted for multiple comparisons designed to locate the significant effects of a factor. A correlational analysis, using Pearson correlation coefficient, was performed to test for age or years of practice effects on the fNIRS parameters. Significant correlations would be included as covariates in an ANCOVA analysis for the functional biomarkers. A two-tailed *p* < 0.05 was considered significant. The magnitude of the effects was also checked with the *η_p_*^2^ value.

## 3. Results

The main characterization of the study population is summarized in Table 1. The 30 male chess players enrolled in the study and had a mean age of 24.15 ± 12.84 years, ranging from 13 to 55 years. All participants were clinically healthy, with three participants reporting the use of medication (mainly anti-histaminic drugs). Mean chess practice was 14.00 ± 10.30 years (range: 6–43 years) and mean chess competition participation time was 10.92 ± 7.85 years (range: 4–40 years). The mean ELO was 1677 ± 332, and all participants referred to regular chess practicing habits as depicted in Table 1. The majority of the participants attributed the beginning of their chess playing either to school activities (46.7%) or family influence (36.6%). The population was divided according to age into a group of adults (age above 19 years) and adolescents (age between 13 and 19 years). As expected, the adults had a significantly longer chess playing background, although no significant differences were observed concerning chess practicing habits. Similar patterns of playing with digital chess platforms and problem-solving training routines were observed in adults and adolescents.

Regarding the initial levels of motivation towards the tasks of the study, a mean score of 7.67 ± 2.26 was obtained for the entire population, with similar results in the adults (mean score: 7.80 ± 2.54) and the adolescents (mean score: 7.53 ± 2.03; *p* = 0.753). The degree of initial tiredness showed a similar trend, with a mean initial score of 2.30 ± 2.07 in the whole population, and no differences comparing adults and adolescents (*p* = 0.467).

The majority of the players (*n* = 22; 73.3%) were able to solve the low difficulty problem, independently of their age (adults: *n* = 12; adolescents: *n* = 10; *p* = 0.409). Regarding the medium difficulty problem, half of the participants were able to solve them, with no significant differences between the adults and the adolescents (*p* = 0.715). None of the participants were able to find the solution for the high difficulty problem.

Table 2 summarizes the main findings for the functional biomarkers in the whole study population. An overall increase in PFC oxygenation with task difficulty was observed, with a mean Δoxy of 0.78 ± 0.21 μmol/L in the low difficulty task, increasing to 0.91 ± 0.26 μmol/L in the medium difficulty task and 0.98 ± 0.25 μmol/L in the high difficulty task (F = 8.782; *p* < 0.001; *η_p_*^2^ = 0.232). Considering the four defined ROIs, significant changes in all four biomarkers were observed only over the L-PFC, showing an increase in ∆HbO2, Δoxy, and ΔHbT with increasing task complexity. These changes were followed by a significant decrease in ∆HHb, indicating a higher activation in the L-PFC as a function of the difficulty of the problem-solving tasks. The aforementioned results explain the significant changes observed when comparing hemispheric contributions according to the task difficulty, with significant changes observed for the left hemisphere region of the PFC (L-PFC and LM-PFC; Δoxy F = 10.896; *p* < 0.001; *η_p_*^2^ = 0.273) but not for the right hemisphere region (R-PFC and RM-PFC; Δoxy F = 1.637; *p* = 0.203; *η_p_*^2^ = 0.053).

**Figure 3:**
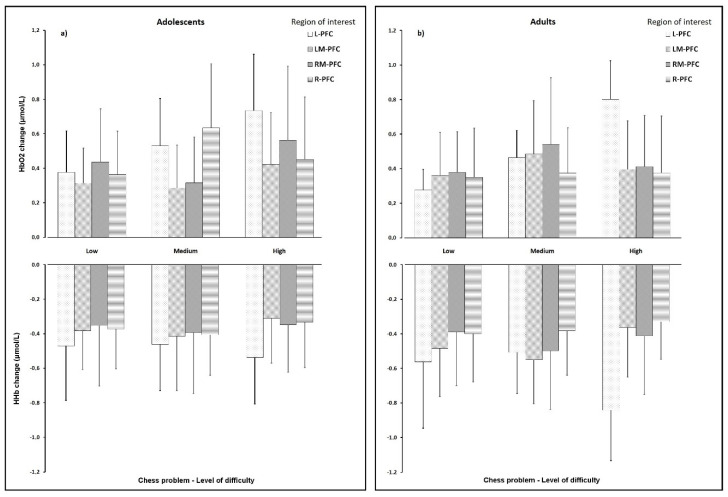
Relative changes in oxygenated hemoglobin (HbO2) and deoxygenated hemoglobin (HHb) in the adolescents group ((**a**) panel) and in the adults group ((**b**) panel), according to the level of difficulty of the chess-based problem-solving tasks. L-PFC—left prefrontal cortex; R-PFC—right prefrontal cortex; LM-PFC—left medial prefrontal cortex; RM-PFC—right medial prefrontal cortex.

Considering the main findings for the functional biomarkers in the adolescents and the adults, different patterns of oxygenation were depicted as demonstrated in Figure 3. A significant Task difficulty*ROI*Group interaction was observed regarding the ∆HbO2 (F_interaction_ = 2.580; *p* = 0.020; *η_p_*^2^ = 0.084) and the ∆oxy (F_interaction_ = 2.345; *p* = 0.034; *η_p_*^2^ = 0.077), but not for the ∆HHb and the ∆HbT. In both groups, significant changes were observed in the L-PFC, with an increase in ∆HbO2, Δoxy, and ΔHbT and a decrease in ∆HHb with increasing task complexity. A significant effect was also observed for the ∆HbO2 in the medium level chess problem, with the adolescents presenting significant greater relative change over the R-PFC when compared with the adults (*F* = 4.808; *p* = 0.004; *η_p_*^2^ = 0.147). No significant changes were observed in the low-level chess problem, but differences emerged with increasing complexity of the task, mainly located in the R-PFC and the dorsolateral regions (both LM- and RM-PFC) for the medium level chess problem, and in the L-PFC for the high-level chess problem. In the medium level chess problem, the adolescents presented higher relative changes in Δoxy over the R-PFC and smaller relative changes in Δoxy over the dorsolateral regions of the PFC. In the higher complexity task, the L-PFC responded more in the adults, leading to greater relative change in the Δoxy as compared with the adolescents. No significant differences were observed between groups in what concerns the overall PFC oxygenation levels in the three experimental conditions. To test for age or years of practice effects, we performed a correlational analysis of the fNIRS parameters and these two covariates. A significant (weak to moderate) correlation was found only between age and the LM-PFC changes in HbO2 (*r* = 0.371; *p* = 0.044), HHb (*r* = −0.378; *p* = 0.039), and Oxy (*r* = 0.449; *p* = 0.013). Consequently, we performed an ANCOVA analysis for the functional biomarkers, with age as covariate, and no substantive changes were observed in the factorial results regarding the ∆HbO2 (F_interaction_ = 2.286; *p* = 0.038; *η_p_*^2^ = 0.078) and the ∆oxy (F_interaction_ = 3.421; *p* = 0.003; *η_p_*^2^ = 0.112), although a significant task difficulty*ROI*Group interaction was depicted for the ∆HHb (F_interaction_ = 2.590; *p* = 0.020; *η_p_*^2^ = 0.088) when age was included as covariate in the model.

## 4. Discussion

The aim of this study was to evaluate and compare the dynamics of the PFC activation during three chess-based problem-solving tasks of increasing level of difficulty in competitive adult and adolescent chess players. Several main findings emerged. First, the activation of the PFC, measured by fNIR spectroscopy, was increased with more demanding chess problems in both adolescent and adult chess players. Second, differences in the patterns of PFC activation were found between adult and adolescent chess players. Adult chess players showed greater relative changes in blood oxygenation over the dorsolateral regions of the PFC and greater activation of the L-PFC during the high difficulty problem, whereas the adolescent chess players showed a greater relative change in the ∆oxy in the R-PFC in the medium level task. However, an alternative explanation could emerge. In this regard, since the performance is the same in the two groups, it could be that younger participants exhibited lower mental effort in the high level condition. Moreover, adults could be more engaged in the high level problem-solving task than young chess players (less motivated than adult chess players). In this line, a lower PFC activation when an individual drops the task has been found in fNIRs studies [38,39].

Considering the factorial profiles of changes in oxyhemoglobin and deoxyhemoglobin, a more efficient management of cognitive resources is apparent in adult chess players, regardless of the overall level of PFC activation. Interestingly, these differences in PFC activation had no consequences in the overall performance. These results have a significant relevance in the field of chess since fNIR spectroscopy could be used to determine the cognitive load of a task as well as to test if a training could change the efficiency of the PFC. In addition, future studies should use this technology in older adults or special populations to evaluate the effects of therapies in the PFC activation.

In order to study the PFC activation, we employed fNIR spectroscopy to measure the relative changes in HbO2 and HHb during the chess tasks. Changes in HbO2 have been considered as the most sensitive parameter to measure activity-dependent changes in regional cerebral blood flow [40,41,42] and is also particularly sensitive to mental workload variations [37,42]. Our main findings are consistent with the involvement of the PFC during the experimental tasks, increasing its activation following the increase in the complexity of the experimental tasks. Furthermore, the defined ROIs in the PFC showed different contributions as a function of the difficulty of the chess problems, particularly in the L-PFC in which a progressively higher activation was identified through the four considered biomarkers with increasing level of difficulty. The increased activation of the PFC for increasingly more demanding chess problems was observed both in adults and adolescents, although the level of activation in the considered ROIs was different particularly in the medium and high difficulty level. The adult chess players showed greater relative changes in blood oxygenation over the dorsolateral regions of the PFC and greater activation of the L-PFC during the high difficulty problem as compared with the adolescents, which showed a greater relative change in the ∆oxy in the R-PFC in the medium level task. Notwithstanding, these between-group differences in the PFC activation had no consequence in the overall performance during the tasks, since no differences were observed between the groups when comparing the level of success solving either experimental scenario.

The enrollment of the PFC in complex tasks has been widely demonstrated [1,2,3,4,5,6,7,9] and was also observed in our study. Furthermore, the dorsolateral region of the PFC has been shown to play a crucial role in the overall coordination of several cognitive resources which are necessary during problem-solving tasks, such as the temporal organization of behavior, language, and reasoning [5], the definition and coordination of plans for action [6], and the flexibility to the environmental demands [7]. This PFC region was mostly active in the chess players during the experimental tasks, and particularly the L-PFC was quite sensitive to changes in the cognitive load as expressed by the difficulty of the problem, with increasing activation following the increase in the complexity of the tasks.

The differences in the patterns of PFC activation in adult and adolescent players could express the interaction of both brain maturation and level of expertise or experience. In fact, previous research identified the recruitment of different psychological functions and the activation of different brain areas or different magnitudes of activation of the same brain areas during chess-related activities in expertise versus novice players [10,14,15,16]. This is in line with our findings which highlight the existence of significant differences in the PFC dynamics during chess-based problem-solving tasks comparing the adult with adolescent chess players. This could confirm that the differences in the PFC detected by previous studies [18] also affect the functioning of the PFC. Curiously, the different patterns of PFC activation were not followed by differences in terms of the overall performance during the task, and, therefore, the differences in brain dynamics over the PFC could merely translate into greater underlying efficiency in the adult players. This could be connected with the chunking theory and its augmented theory, the template theory [43]. Chase and Simon [44] proposed that Masters access information in long-term memory rapidly by recognizing familiar constellations of pieces on the board, the patterns acting as cues that trigger access to the chunks. This could be the reason why cognitive processes are more efficient in adults than in adolescent chess players. However, future studies should investigate this hypothesis.

This study has several limitations that should be considered. The small number of participants is a significant aspect, particularly on the between-group comparison, even though the results were consistent in the most relevant outcomes considered in the study. We did not analyze the temporal changes in PFC oxygenation during the tasks, and, therefore, the time course of hemodynamic changes during the problem-solving tasks was not considered for the analysis. Considering that the fNIRS system used in this research does not integrate short channels-based technology, we cannot exclude the possibility of picking extra-cerebral oxygenation signals. Such limitation could have been obviated through the use of task replications, and, therefore, further studies should include replications in the experimental design. The difference in the ELO score and overall chess experience between groups could also explain some of the observed differences at the group level and should be addressed in future research. Due to the heterogeneity of our sample, future studies should explore the PFC activation during problem-solving tasks in different levels of expertise (novice vs. expert paradigm) in age-matched groups.

## 5. Conclusions

To the best of our knowledge, this is the first study addressing the dynamics of the PFC activation during chess-based problem-solving tasks with fNIR spectroscopy. The PFC dynamics differ in adults and adolescents, corresponding to a more efficient cortical organization in the adult players for the same overall level of performance. Furthermore, we demonstrated the participation of the PFC during complex chess problem tasks, with the L-PFC responding with increasing activation to the increasing level of difficulty of the tasks and corresponding cognitive load in both adults and adolescents.

Our findings contributed to a better understanding of the PFC resources mobilized during the handling of complex problems associated with chess-playing, also adding evidence to the understanding of the neural substrate underlying overall human problem-solving mechanisms. Moreover, revealed differences in the PFC functioning between adults and adolescents during high cognitive demand tasks should be investigated in future studies. Further studies should also include the continuous measurement of fNIRS-based biomarkers to allow for the examination of spontaneous hemodynamic fluctuations (as in Verdière et al.’s [45] study), as well as the adoption of more sophisticated NIRS technologies, such as high-definition near-infrared spectroscopy or diffuse optical tomography. Studying the functional correlates of unsuccessful moves during chess playing could also add novel insights, and connectivity metrics could also support the study of hemispheric interplay during complex chess problem tasks, contributing to a better understanding of cortical dynamics in chess playing.

## Figures and Tables

**Table 1:** Characterization of the participants according to age, chess practicing habits, and individual ELO.

	Total (*n* = 30)	Adults (*n* = 15)	Adolescents (*n* = 15)	*Ƥ*
**Age (years)**	24.2 ± 12.8	32.7 ± 13.5	15.6 ± 1.7	<0.001
**Chess playing (years)**	14.0 ± 10.3	19.6 ± 12.2	08.4 ± 2.0	0.003
**Chess competition (years)**	10.9 ± 7.8	14.7 ± 9.6	07.2 ± 2.2	0.010
**Chess practicing habits**				
**Days/week**	2.5 ± 2.2	2.5 ± 2.3	2.5 ± 2.2	0.968
**Hours/Day**	1.7 ± 1.3	1.8 ± 1.3	1.6 ± 1.4	0.689
**Hours/week**	5.2 ± 6.3	6.1 ± 7.3	4.4 ± 5.1	0.470
**ELO**	1677 ± 332	1825 ± 249	1529 ± 347	0.012
**Number of problem-solving chess tasks solved**				
**Low difficulty**	22 (73.3%)	12 (80%)	10 (66.7%)	0.409
**Medium difficulty**	15 (50%)	7 (46.7%)	8 (53.3%)	0.715
**High difficulty**	0 (0%)	0 (0%)	0 (0%)	1.000

**Table 2:** Prefrontal cortex dynamics according to the variation of the functional biomarkers in the three problem-solving chess tasks.

		Low Difficulty	Medium Difficulty	High Difficulty	*F*	*P*	*η_p_* ^2^
**∆HbO2** **(μmol/L)**	L-PFC	0.33 ± 0.19	0.50 ± 0.22	0.77 ± 0.28	71.656	<0.001	0.712
	R-PFC	0.36 ± 0.26	0.50 ± 0.34	0.41 ± 0.34	1.547	0.221	0.051
	LM-PFC	0.34 ± 0.22	0.38 ± 0.29	0.41 ± 0.29	0.753	0.476	0.025
	RM-PFC	0.41 ± 0.27	0.43 ± 0.34	0.49 ± 0.37	0.563	0.572	0.019
**∆HHb** **(μmol/L)**	L-PFC	−0.51 ± 0.35	−0.48 ± 0.25	−0.69 ± 0.32	3.901	0.026	0.119
	R-PFC	−0.39 ± 0.25	−0.39 ± 0.24	−0.33 ± 0.24	1.049	0.357	0.035
	LM-PFC	−0.43 ± 0.25	−0.48 ± 0.29	0.34 ± 0.27	2.409	0.099	0.077
	RM-PFC	−0.37 ± 0.33	−0.45 ± 0.34	−0.38 ± 0.31	3723	0.490	0.024
**∆HbT** **(μmol/L)**	L-PFC	−0.19 ± 0.42	0.01 ± 0.26	0.08 ± 0.35	5.865	0.005	0.168
	R-PFC	−0.3 ± 0.35	0.11 ± 0.27	0.09 ± 039	1.325	0.274	0.044
	LM-PFC	−0.10 ± 0.29	−0.10 ± 0.32	0.07 ± 0.36	2.727	0.074	0.086
	RM-PFC	0.04 ± 0.42	−0.02 ± 0.40	0.11 ± 0.39	0.939	0.397	0.031
**∆oxy** **(μmol/L)**	L-PFC	0.84 ± 0.38	0.98 ± 0.39	1.50 ± 0.48	23.777	<0.001	0.451
	R-PFC	0.74 ± 0.38	0.90 ± 0.53	0.74 ± 0.45	1.150	0.229	0.049
	LM-PFC	0.77 ± 0.39	0.87 ± 0.49	0.75 ± 0.43	0.898	0.413	0.030
	RM-PFC	0.78 ± 0.43	0.87 ± 0.56	0.87 ± 0.56	0.438	0.648	0.015

∆HbO2—variation in oxyhemoglobin; ∆HHb—variation in deoxyhemoglobin; ∆HbT—variation in total hemoglobin; ∆oxy—difference between oxyhemoglobin and deoxyhemoglobin; L-PFC—left dorsolateral prefrontal cortex; R-PFC—right dorsolateral prefrontal cortex; LM-PFC—left medial prefrontal cortex; RM-PFC—right medial prefrontal cortex.
